# FluConvert and IniFlu: a suite of integrated software to identify novel signatures of emerging influenza viruses with increasing risk

**DOI:** 10.1186/s12859-020-03650-y

**Published:** 2020-07-18

**Authors:** Chin-Rur Yang, Chwan-Chuen King, Li-Yu Daisy Liu, Chia-Chi Ku

**Affiliations:** 1 Institute of Immunology, College of Medicine, National Taiwan University (NTU), 1 Jen-Ai Road Section 1, Taipei, 10051 Taiwan, Republic of China; 2 Institute of Epidemiology and Preventive Medicine, College of Public Health, NTU, Taipei, 10055 Taiwan, Republic of China; 3 Division of Biometry, Department of Agronomy, NTU, Taipei, 10617 Taiwan, Republic of China; 4 Department of Agronomy, National Taiwan University, No. 1, Section 4, Roosevelt Rd, Taipei, 10617 Taiwan

**Keywords:** Highly pathogenic avian influenza viruses, H5N2, Viral and immunological informatics, Risk assessment, Pandemic potential

## Abstract

**Background:**

The pandemic threat of influenza has attracted great attention worldwide. To assist public health decision-makers, new suites of tools are needed to rapidly process and combine viral information retrieved from public-domain databases for a better risk assessment.

**Results:**

Using our recently developed FluConvert and IniFlu software, we automatically processed and rearranged sequence data by standard viral nomenclature, determined the group-related consensus sequences, and identified group-specific polygenic signatures. The software possesses powerful ability to integrate viral, clinical, and epidemiological data. We demonstrated that both multiple basic amino acids at the cleavage site of the HA gene and also at least 11 more evidence-based viral amino acid substitutions present in global highly pathogenic avian influenza H5N2 viruses during the years 2009–2016 that are associated with viral virulence and human infection.

**Conclusions:**

FluConvert and IniFlu are useful to monitor and assess all subtypes of influenza viruses with pandemic potential. These programs are implemented through command-line and user-friendly graphical interfaces, and identify molecular signatures with virological, epidemiological and clinical significance. FluConvert and IniFlu are available at https://apps.flutures.com or https://github.com/chinrur/FluConvert_IniFlu

## Background

The emergence of novel H5N1 avian influenza virus (AIV) in 1997 resulting in fatalities in humans has raised global concern [1]. As of May 8, 2020, a total of 861 human infections and 455 deaths caused by H5N1 infection had been reported [2]. Thereafter, the re-emergence of highly pathogenic avian influenza (HPAI) A H5Ny subtypes that cause widespread infections in poultry farms and in wild birds since 2003 has greatly attracted public health attention. Interestingly, the H5 AIVs in Asia have evolved faster, having higher viral diversity, greater inter-species transmission, and broader host range than those in Europe and the Americas [3]. Understanding the viral factors which determine the pathogenicity of H5 AIV by timely integration of virological, immunological and epidemiological information will be helpful to establish effective prevention and control measures to minimize future pandemic threats.

The immediate release of the genetic sequences of influenza A viruses combined with collections of tools established for analyzing all types and subtypes of influenza viral sequences have greatly advanced our understanding of the evolution of circulating viruses and their potential risk to animal and human health [4]. Given the fact that multiple mutations across gene segments of influenza viruses can exist and the genomic stability might be influenced by a particular mutation over time [5], new suites of tools are needed to integrate these databases for a better alignment of virological, epidemiological and clinical data in a real-time manner.

Several public-domain databases are available for collecting influenza genetic and epidemiological information. They include: (1) National Center for Biotechnology Information Influenza Virus Database (NCBI-IVD) [6], (2) Global Initiative on Sharing All Influenza Data (GISAID-EpiFlu) [7], and (3) Influenza Research Database (IRD) [8]. While NCBI-IVD provides the complete influenza viral sequences of gene segment across a wide range of years, GISAID-EpiFlu is recognized as a compelling mechanism for rapid sharing of partial or incomplete influenza viral sequences [9]. As for IRD, it contains human and mammalian influenza surveillance data as well as human clinical data associated with viruses, linking host surveillance data to well-characterized virus strains [8].

In this paper, we reported on development of a new suite of integrated software including FluConvert and IniFlu for data processing and analysis. FluConvert provides a series of automated packages to efficiently rearrange genetic data based on standard viral nomenclature [10] and translate the nucleotide sequences into three possible polypeptides from 0, + 1, and + 2 open reading frames (ORF) after performing simultaneous multiple sequence alignments. For IniFlu, it is programed to automatically select the correct ORF encoded from corresponding gene segment as well as the spliced isoforms (e.g. NS1, NS2 of NS gene; M1, M2 of M gene). Possible accessory proteins (e.g. PB1-N40, PB2-S1, M42) that have been reported in the literatures [11–13] can also be selected by IniFlu. The capability of IniFlu that integrates viral genetic information into clinical and epidemiological surveillance data with high efficiency provides a rapid comparison of variations in viral sequences with epidemiological significance. To this end, we provide the results from analysis of H5N2 HPAI viruses defined by the presence of the hallmark amino acid motif (XRRKRR) at the cleavage site between HA1 and HA2 domains [14]. In addition to these multiple basis amino acid residues in the HA, we demonstrate that several other amino acid substitutions across different gene segments of H5N2 avian influenza viruses could be associated with the viral virulence and mammalian infections based on IniFlu-generated polygenic HPAI consensus signature. This suggests that the data analysis platform we report here will be useful to identify novel mutations for risk assessment of AIVs with potential threat to animal and human health.

## Methods

### Installation of FluConvert and IniFlu

**Fig. 1:**
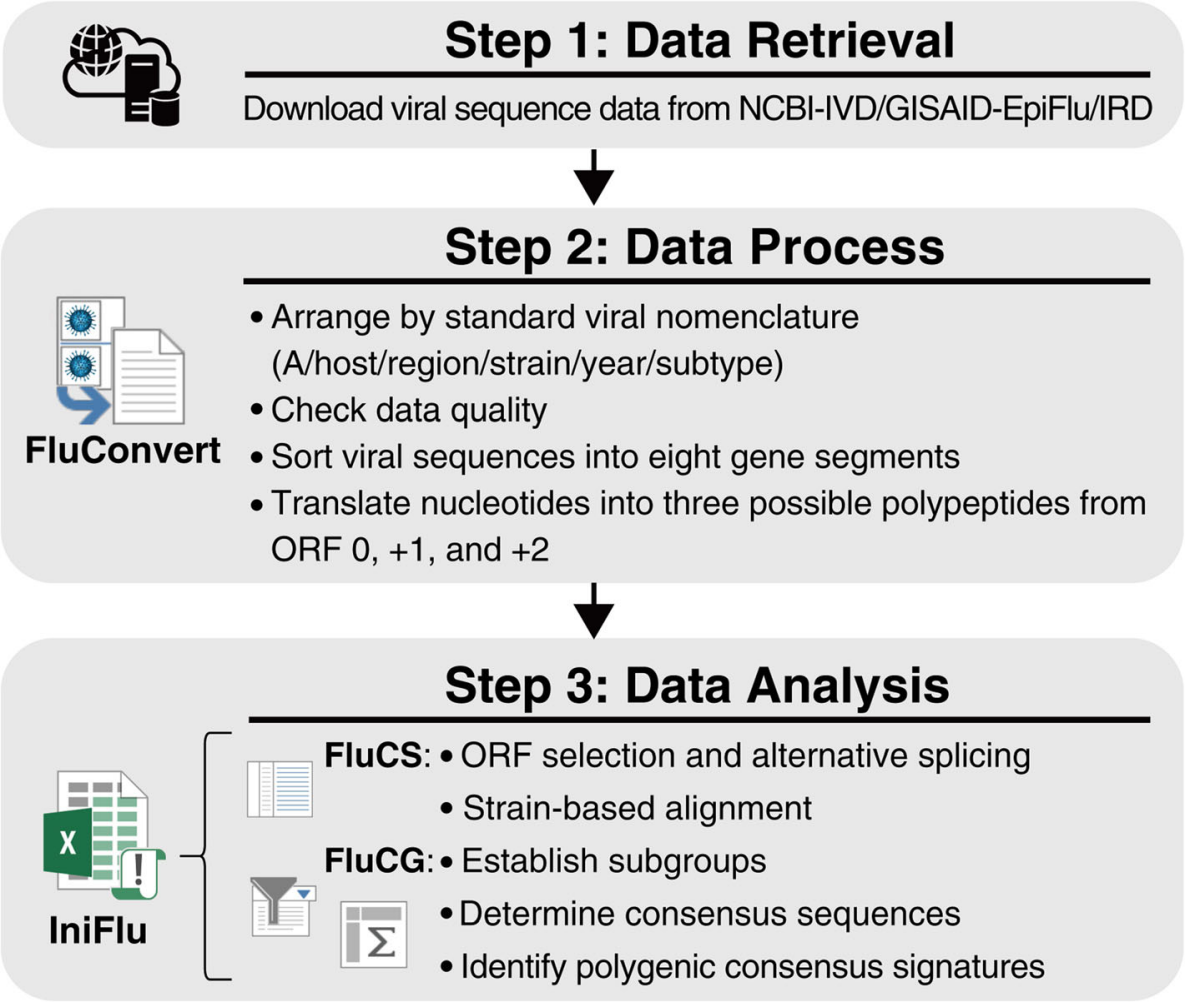
Workflows of data analysis executed by FluConvert and IniFlu. The stepwise processes performed by FluConvert and IniFlu to identify novel signatures of emerging influenza viruses with increasing risk are described as follows. Step 1: Viral sequences are obtained from the three databases (NCBI-IVD, GISAID-EpiFlu, and IRD). Step 2: FluConvert rearranges viral strains by viral nomenclature and ensures data quality. Viral sequences are further sorted into eight gene segments and translated into amino acid sequences. Step 3: The module FluCS of IniFlu performs strain-based alignments of FluConvert-processed viral amino sequences. The module FluCG of IniFlu regroups viral strains with epidemiological significant and computes a consensus sequence for each subgroup. Finally, the subgroup-specific unique polygenic amino acid signatures can be simultaneously identified (see details in Fig. 3)

Both FluConvert and IniFlu are available for free download at https://apps.flutures.com or https://github.com/chinrur/FluConvert_IniFlu. The programs can be automatically installed in a desktop computer after the user perform execution files which is found in downloaded folders. The operating system of the computer requires Microsoft Windows 10 (version 1903 or later version) equipped with Microsoft Office 365 or Office Excel 2016 (or later version, 64-bit) for installation and Java 6 (or later, 64-bit) to perform each software. When open FluConvert, the user will be asked to import data which have been pre-downloaded from NCBI-IVD or GISAID-EpiFlu (Step 1 of Fig. 1) following instructions provided on the website. It will take minutes to hours to complete the process depending on the quantity of data entry. Once FluSeed Dataset has been processed by FluConvert (Step 2 of Fig. 1), IAV sequences can be analyzed by IniFlu (Step 3 of Fig. 1). We also include a detail step-by-step user’s guide in Readme which can be found at https://apps.flutures.com website.

### FluConvert: a tool to process downloaded sequences

FluConvert automatically processes downloaded sequence files (*.FASTA) using the command-line interface (CLI) by batch (shell) scripts operated in a Microsoft Windows environment. It consequently performs (1) name and quality checking for downloaded sequences, (2) separation of sequences into eight gene segments, (3) multiple alignment of DNA sequences within clusters, (4) translation of DNA sequences into three possible polypeptides from ORF 0, + 1, and + 2, and (5) multiple alignment of amino acid sequences within clusters. The functions of FluConvert are to unify the arrangement of genetic information and then to convert nucleotide sequences of cDNA to amino acid sequences for multiple alignment at the protein level.

#### Arrangement and quality checking for downloaded sequences

All sequences downloaded from the NCBI-IVD and the GISAID-EpiFlu databases are rearranged according to the standard influenza viral nomenclature in the order of type, host, region, strain, year, and subtype within the parentheses [10]. Secondly, rearranged sequences are inspected, and the gene segments are deleted when they met any conditions in the “excluding list” generated for quality checking. Entries retrieved from NCBI-IVD and GISAID-EpiFlu databases were deleted according to “excluding list” to remove duplicates, incomplete sequences, or those with error information. Downloaded entries that are later saved to FluSeed Dataset have never been modified or corrected for any purposes. This is to ensure that the information remains original and the features of genetic sequences are kept unaltered during FluConvert processing. The three major error conditions of viral sequence information are: (1) lacking complete viral nomenclature, having mixed subtypes, belonging to lab strains or showing errata in public database records, (2) finding duplicate sequence records in any of the public databases, and (3) sequences longer than the expected lengths for different segments (e.g. PB1 > 2500 bp, PB2 > 2500 bp, PA > 2400 bp, HA > 1900 bp, NP > 1700 bp, NA > 1600 bp, M > 1150 bp, and NS > 1050 bp), or having redundant sequences or those containing more than 60 unknown nucleotides (denoted as ‘n’). Finally, all the sequences that had passed the excluding list’s quality check without entering the excluding list were used to create a new dataset called the “FluSeed Dataset” and subjected to IniFlu analysis.

As noted, entries retrieved from these public domain databases have never been modified or corrected after downloading. This ensures to keep information original and features of genetic sequences are not lost during FluConvert processing. Moreover, FluSeed Dataset is used for IniFlu analysis and has never been intended to make publicly accessible.

#### Multiple sequence alignment and amino acid translation

The genome of influenza A virus contains eight RNA segments. Therefore, FluConvert first divides the genetic sequences in FluSeed Database into eight clusters by the MAFFT multiple sequence alignment program (version 7.429) with fast Fourier transform [15]. All sequences in each of the eight gene segments are then translated into three possible polypeptides from ORF 0, + 1, and + 2 by EMBOSS Transeq (version 6.5) [16]. Nucleotide sequences and amino acid sequences in each of the eight gene segments in the FluSeed Database are subject to multiple alignment by MAFFT again with different optimizing parameters based on sequence lengths and the numbers of viral strains or files [i.e. L-INS-i (accurate) for alignment of <∼200 viral strains/files; FFT-NS-2 (fast) for alignment of <∼30,000 viral strains/files to obtain maximal efficiency; and PartTree (fast) for alignment of > ∼ 30,000 viral strains/files] [17]. Results of sequence alignments from the same ORF were saved as comma-delimited (csv) text files.

### IniFlu: a viral information viewer and analyzer

IniFlu, a Visual Basic Application (VBA) program for Microsoft Office Excel 2019 worksheet, has a user-friendly graphical interface (GUI) to combine viral information, amino-acid sequences and epidemiological data for further analyses. IniFlu has two modules, “FluCS” (which stands for “Flu Cross-Segment alignment”) and “FluCG” (which stands for “Flu Comparative Grouping”). FluCS matches the aligned sequences according to the standard viral nomenclature of the strains after encoding protein from ORF selection and alternative splicing. FluCG visualizes different epidemiologically specific (such as time-, area-, host-, age-, gender-specific) consensus signatures obtained (shown in Fig. 4), providing not only the clinical information of the viral sequences but also their epidemiological characteristics.

#### FluCS: strain-based amino acid sequence alignment

**Fig. 2:**
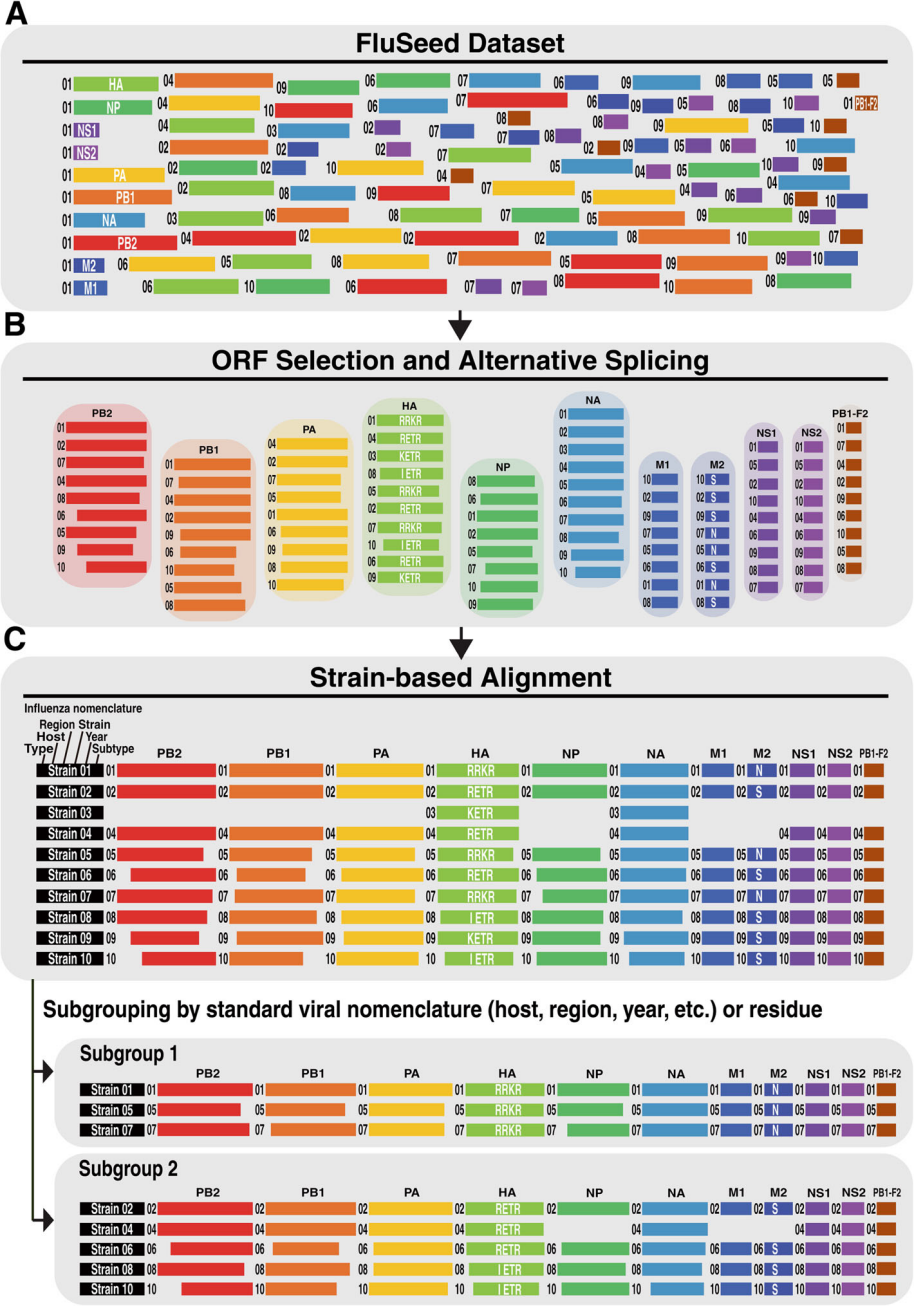
Schematic diagram of strain-based alignment approach. Influenza viral sequences are aligned by FluCS as follows: **a** The FluSeed Dataset is constructed by quality-checked and rearranged viral sequences. Blocks in different colors represent ten viral segments. The size of each block corresponds to the length of the viral sequence originally retrieved. Blocks in any color tagged with the same Arabic numbers are identified as the same strain. **b** Rearranged viral sequences are sorted into 11 protein clusters based on gene segments and well aligned within the cluster. Aligned sequences are subjected correct ORF into PB2, PB1, PA, HA, NP, NA, M1, NS1, and PB1-F2. M2 and NS2 are alternatively spliced proteins from M and NS ORF mRNAs, and respectively. **c** Delineated viral amino acid sequences are easily aligned based on standard influenza viral nomenclature. The analysis platform provides benefits for multi-layer subgrouping based on epidemiological significance

FluCS groups the amino-acid sequences of each gene segment according to FluSeed Dataset (Fig. 2a). FluCS is also programed to automatically select the correct ORF of each viral protein as well as the alternatively spliced isoforms. Accessory proteins, e.g. PB1-N40, PB2-S1 and M42 [11–13] can be assigned to the viral segment group of PB1, PB2, and M, respectively. PB1-F2 which is translated by a second ORF in the + 1 frame [11, 18] is selected and assigned to an independent group. As a result, a total of 11 viral segment groups (PB1, PB2, PA, HA, NA, NP, M1, M2, NS1, NS2, and PB1-F2) is established for strain-based alignment (Fig. 2b, c). The position of each residue is numbered based on the first methionine residue of that gene segment determined by FluCS (e.g. HA of H5N2 subtype is numbered by H5 numbering system) [19].

#### FluCG: comparative sequence analysis

FluCG chooses the most representative amino acid at each residue of a particular gene segment by computing the most frequent amino acid among all strains within the studied subgroup. If there are more than two amino acids occurring at the same frequency, one is chosen by alphabetic order. Residues that appear at stop codons or deleted codons are marked. Through this process, the consensus sequence can be created for a particular subgroup [20]. The unique residues in each subgroup can also be identified by aligning two consensus sequences and are called “consensus signatures”. All the unique amino acids appearing at each residue of the consensus signature are thoroughly examined and compared to verify the unique amino acid is present only in the particular subgroup. Finally, all possible 20 amino acids, stop codons, and deletions are all examined and presented in a substitution table (as Fig. 5).

## Results

### Influenza viral sequences and data processing

The genetic sequences and epidemiological information of influenza viruses in one public domain database are not properly linked to the other. To maximize the information coverage for a particular AIV subtype for further analysis, we have developed the FluConvert program to combine all data available from these databases and automatically process them in one format. Viral sequences that had passed quality check after excluding incomplete or erroneous ones to ensure correct genetic information are used for constituting the FluSeed Dataset. Sequences in the FluSeed are subsequently rearranged to standard nomenclature in the order of influenza virus of type/host/region/strain/year (HxNy subtype) and segregated into eight gene segments. Amino acid sequences are translated from nucleotide sequences for alignment (Fig. 1).

### Viral strain-based sequence alignment

Continuous mutations in HPAI A (H5) viruses have been attributed to outbreaks at poultry farms and sporadic human infections [21]. Since mutations can occur across several gene segments in the genome of AIVs, multiple alignments for all viral strains based on the subgroup of interest (i.e. host, region, year, a particular residue, etc.) rather than by gene segment (i.e. HA, NA, PB2, etc.) will be useful to identify multiple amino acid types associated with viral pathogenicity in animals or the potential risk for human infections. To achieve the goal, we have developed the IniFlu platform to integrate the processed viral sequences, clinical and epidemiological information into the FluSeed Dataset. IniFlu can function to present all the information imported from FluSeed in worksheet outputs for visual cross-segment examinations simultaneously. Once all of the viral strain information is correctly arranged by FluCS, strain-based alignment can be quickly performed as illustrated in Fig. 2.

### Identification of polygenic consensus signatures

**Fig. 3:**
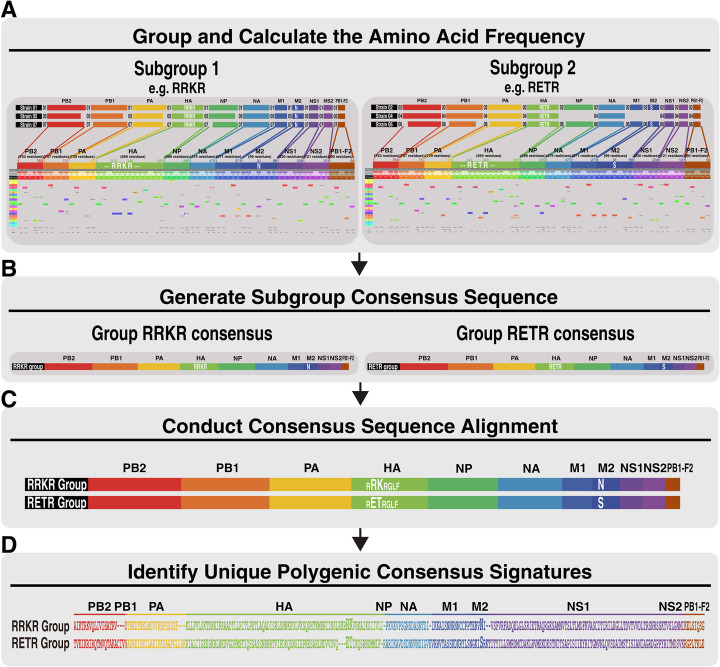
Identification of group-specific polygenic consensus signatures by FluCG. The polygenic consensus signatures are determined as follows: **a** FluCG groups viral strains into different subgroups (e.g. RETR and RRKR groups). The consensus sequence of each subgroup is determined by computing a table of 20 amino-acid substitutions to choose the most representative amino acid at each position of the whole genome. The stop codon is denoted as “X” and the deleted residue is denoted as “-”. **b** One consensus sequence from each subgroup is determined. **c** FluCG aligns two group consensus sequences and identifies the signature that is unique, to distinguish between the two consensus sequences. **d** Text in different colors represents different proteins that compose the polygenic consensus signatures

Genetic evolution of zoonotic influenza viruses is a polygenic trait. Amino acid substitutions or mutations at species-associated signature positions may increase viral pathogenicity or mammalian adaptation in a broader host range [22]. Since such mutations are not limited to one gene and can simultaneously occur in multiple gene segments, identification of the polygenic consensus signatures for a particular subgroup of viral strains offers an opportunity to monitor the changing landscape of AIVs over time with epidemiological significance. The module FluCG of IniFlu can quickly group viral strains into different subgroups and deduce the consensus sequence of each subgroup by computing and determining the most representative (i.e. most frequent) amino acid at each position of the whole genome, which can differentiate between the compared subgroups. All unique amino acid residues represented in the subgroup constitute the polygenic consensus signature (Fig. 3).

### Polygenic consensus signatures of the HPAI H5N2 viruses

Duplicate entries of downloaded influenza viral genetic sequences could possibly occur when (1) the entry was submitted to both NCBI-IVD and GISAID-EpiFlu databases, (2) the entry was submitted to one database more than once, or (3) data entries imported from NCBI-IVD co-existed in GISAID-EpiFlu database. To obtain the accurate count of H5N2 virus strains downloaded from different public domain databases, FluConvert is programmed to automatically remove duplicate entries. Only one copy of the gene segment of a single virus strain is kept in FluSeed Dataset.

As of July 1, 2017, the H5N2 FluSeed Dataset was comprised of a total of 6746 (6443 + 303 = 6746) unique gene segments that belong to 1151 (1099 + 52 = 1151) H5N2 viruses. Amongst which, 6443 gene segments of 1099 H5N2 strains were downloaded from NCBI-IVD and 303 segments of 52 H5N2 strains were downloaded from GISAID-EpiFlu, respectively. Qualified genetic sequences were rearranged by FluConvert to unify the nomenclature format. Corresponding epidemiological information and clinical data for each strain were integrated through the IniFlu platform. Since several studies have demonstrated that the presence of multiple basic amino acids at the cleavage site between HA1 and HA2 junctional sequence is a hallmark for increasing viral pathogenicity and virulence in the avian host and humans [14], we compared the molecular signature in the H5N2 viral strains with (RRKR group) or without (RETR group) polybasic residues at the cleavage site in the HA gene. The earliest record of H5N2 viruses was reported in 1972 and all of the 470 strains isolated during 1972–2008 appeared to have RETR sequence motif at the HA cleavage site. H5N2 viruses with the RRKR sequence motif in the HA only appeared after year 2009. To avoid bias towards evolutionary perspective, we excluded H5N2 viruses that were isolated before 2008 and only kept the H5N2 viruses isolated from year 2009 to 2016 in the H5N2 FluSeed Dataset for consensus signature analysis of both groups. As a result, there were 165 strains of H5N2 viruses with RETR marker and 138 strains with RRKR marker.

The consensus sequence analysis by FluCG identified 247 unique amino acid residues differentially presented between RRKR and RETR groups in the whole genome of H5N2 AIVs. Since these unique residues were present across several viral segments, we wanted to know which gene segment might present the most unique residues that may distinguish H5N2 viruses with the REKR marker from those RRKR. Table 1 shows the frequencies of the characteristic substitutions that occurred at a particular gene segment. We found that NS1 had the highest substitutions (*N* = 69, 30%), followed by HA (*N* = 77, 13.53%), and PB1-F2 (*N* = 8, 8.89%). There were much less substitutions in NP (*N* = 1, 0.2%) and PB1 (N = 1, 0.13%) of H5N2 viruses (Table 1).

**Table 1:** The 247 residues differentially occurring between RRKR and RETR consensus signatures are polygenic

	Influenza viral proteins										
	PB2	PB1	PA	HA	NP	NA	M1	M2	NS1	NS2	PB1-F2
**Segment size**^**a**^	763	757	729	569	499	475	271	99	230	121	90
**No. of consensus signatures between groups**^**b**^**(%)**	20 (2.62)	1 (0.13)	23 (3.16)	77 (13.53)	1 (0.2)	18 (3.79)	15 (5.54)	8 (8.08)	69 (30)	7 (5.79)	8 (8.89)

^a^: The H5N2 viral genome is composed of 4603 amino acid residues divided into 11 viral proteins. The size of each segment is indicated by the number of residues as shown

^b^: Unique amino acid residues are identified by comparing the consensus sequences between the two studied subgroups. Numbers shown are the counts of the characteristic residues in each viral protein. The variations in each viral protein are expressed by the percentage of unique residues indicated in the parentheses

**Fig. 4:**
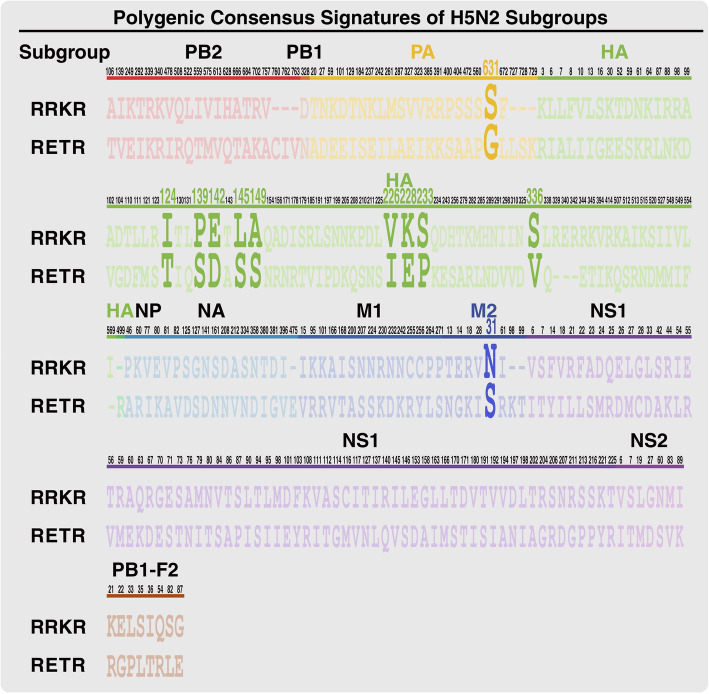
Polygenic consensus signatures of H5N2 RRKR and RETR subgroups. A total of 247 residues across 11 protein segments of H5N2 AIVs obtained from the years 2009 to 2016 are identified from the differences between the RRKR and RETR subgroups. Polygenic consensus signatures for each group are shown by the constellation of the 247 unique amino acid residues across different protein segments (distinguished by colors). The residue positions at each protein are shown as numbers. Of the 247 residues in the consensus signature, 11 evidence-based residues documented in the literature are listed in the enlarged letters with darkened colors

**Fig. 5:**
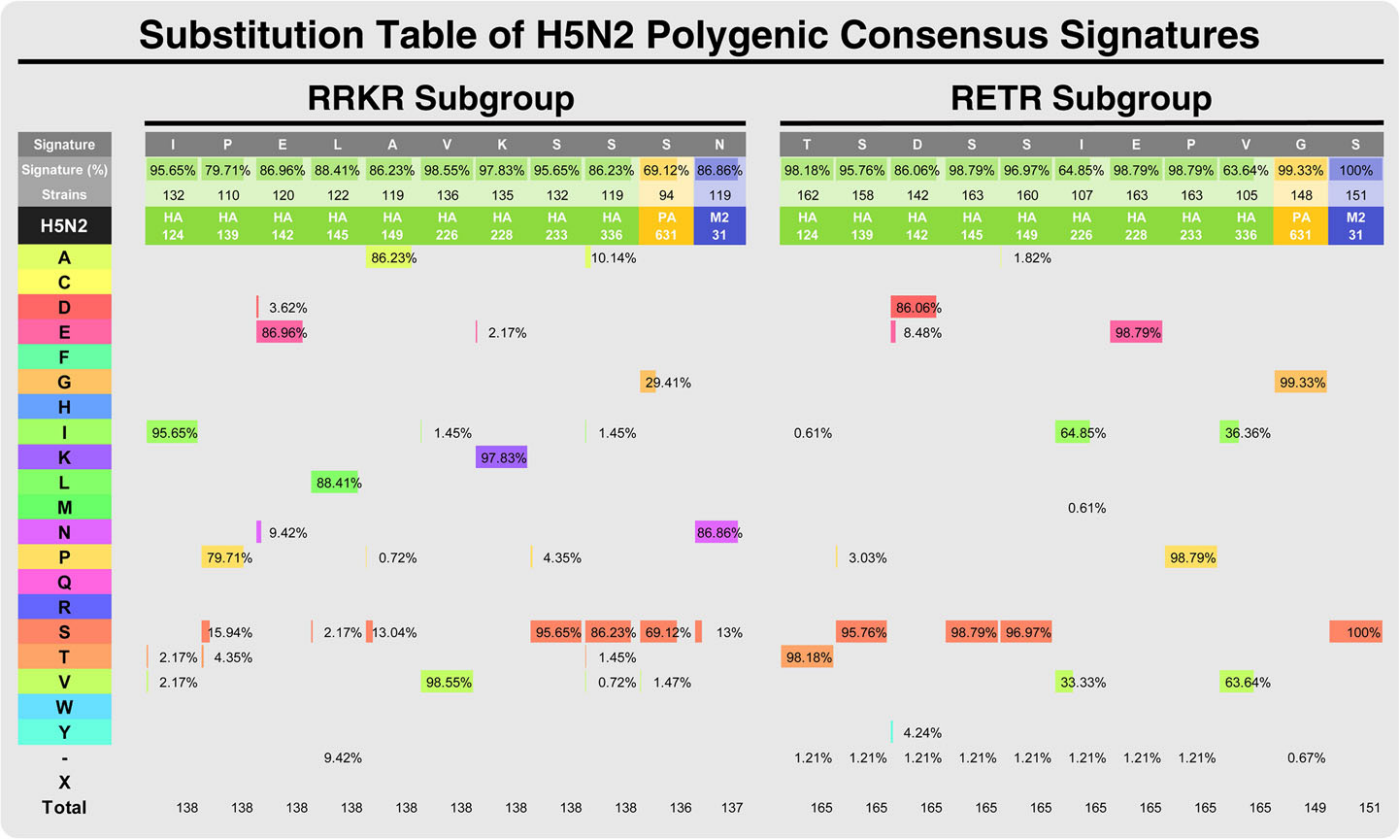
Establishment of polygenic consensus signatures of RRKR and RETR groups of H5N2 AIVs. To further confirm that each residue in the RRKR signature is unique compared with that in the RETR signature, the percentage of the most predominant amino acid (top two rows) at the corresponding position (the 3rd row) derived from the 11 evidence-based residues were re-examined by inspecting the FluCG-generated worksheet. As is shown, the percentages of the predominant amino acids in each signature range from greater than 69.12 to 95.65%

To investigate what substitution at a particular residue or residues could be associated with the RRKR phenotype, the polygenic consensus signatures determined from the constellation of the 247 distinct residues as described in Table 1 were further analyzed (Fig. 4). In search of information on amino acid substitutions in the influenza viruses that are associated with increased viral virulence or drug resistance [23] reported in the public domain database IRD-SFVT (Sequence Feature Variant Types) by IniFlu analysis, we found that substitutions in HA, including T124I, D142E, E228K, P233S, V336S in HA, G631S in PA that are related to increasing pathogenicity [24–26] were present in the RRKR signature. Other variations in the HA of the RRKR signature involved in the increase of α-2.6 receptor binding in mammalian cells such as S139P, S145L, S149A, and I226V [27–30] were also found in our analysis. Notably, the fact that IniFlu identified the substitution of S31N in the M2 of the RRKR signature suggests that H5N2 HPAI may have a decreased sensitivity to amantadine and rimantadine [31] (Fig. 4). All of the unique 11 consensus signatures were re-examined and verified from FluCG-generated substitution table (Fig. 5). Taken together, IniFlu can identify additional substitutions across the gene segments of H5N2 that are highly associated with viral pathogenicity and/or antiviral drug resistance.

## Discussion

Influenza is an important disease in humans and animals. The 13,588-base-pair RNA genome segregated into eight gene segments continues to mutate randomly at 2 × 10^− 6^ mutations per site per infectious cycle [32]. The high activity in the reassortment of segmented influenza viral genes derived from different host species has posed a great threat to public health. Numerous tools have been developed to analyze influenza genetic sequences to monitor the changes and evolution of these viruses over time in nature. In this study, we have added two integrated analysis tools, FluConvert and IniFlu, to the endeavor.

Several analysis tools for IAV genetic sequences are available online to determine antigenic characteristics of IAVs based on the genomic sequences of a particular gene segment and associated epidemiological information. Here we compare a recently published program FluPhenotype [33] with IniFlu. FluPhenotype is a web-based tool. Briefly, IAVs amino acid markers associated with human adaptation, enhanced virulence, and drug resistance, etc. that have been reported in the literatures are captured to the Data list of FluPhenotype. The input genetic sequences of IAVs are mapped with the list and the antigenic characteristics of the IAVs of interest are rapidly determined. FluPhenotype also has the capacity to predict IAV HA subtype and viral hosts based on the input genomic or protein sequences [33]. Although the Data list used in FluPhenotype is reportedly updated every half a year, any newly identified or undefined molecular markers that have not been made available in the literature would not be captured and mapped in a timely manner [33].

In comparison with FluPhenotype, FluConvert is used to sort IAV genetic entries that are downloaded from different public domain databases into eight gene segments based on the name of the gene segment (e.g. PB2, PB1, PA, … etc.). FluConvert subsequently rearranges the information tagged to each entry according to the standard IAV nomenclature in the order of type, host, region, strain, year, and subtype, thereby assigning a unique name to each virus. Therefore, gene segments that have the same name will be grouped as one strain. The capability of FluConvert that determines the correct protein sequences encoded by each viral gene segment and their spliced isoforms as well as accessory proteins results in 11 viral protein clusters in the FluSeed Dataset for strain-based alignment by FluCS.

Since FluCS can align a larger number of viral strains at one time, it saves time on cross-referring of each genetic sequence in NCBI-IVD/GISAID-EpiFlu by accession number. Additionally, the ability of FluConvert to combine information between databases can collect all available influenza genetic data as much as possible by avoiding the exclusion from incomplete information in the depository database. Data in the FluSeed Dataset can be maintained up to date by downloading newly depository of influenza viral genetic data in public domain databases by users.

There are two advantages of IniFlu-performed strain-based alignment and consensus sequence analysis. First, genetic sequences of a viral strain lacking eight complete gene segments can be compared and included for consensus sequence analysis. Second, once the information is properly aligned, sequence data can be easily re-grouped for a generating group-specific consensus sequence. As a result, polygenic consensus signatures composed of unique molecular positions across all gene segments that are associated with a particular phenotype will be determined. As demonstrated from the analysis of the sample H5N2 FluSeed Dataset by comparing the group-specific polygenic consensus signatures between the RRKR and the RETR groups, we identified that at least 247 positions of the total 303 H5N2 AIV strains from 2009 to 2016 were able to differentiate these two groups, and 11 of these substitutions have been experimentally demonstrated for the significance in crossing over between host species (e.g. S139P, S145L, S149A, and I226V in HA) [27–30], antiviral drug amantadine and rimantadine resistance (S31N in M2) [31] or increasing viral pathogenesis (e.g. T124I, D142E, E228K, P233S, and V336S in HA, and G631S in PA) [24–26]. Although there have not been reports of fatal human cases of H5N2, human infection of this AIV subtype have occurred, as documented in seroepidemiological studies [34, 35]. These substitutions together with those residues involved in enhancing receptor binding to mammalian cells [14] have suggested the potential threat to human health caused by H5N2 AIV strains with an RRKR phenotype.

Taken together, we reported the newly developed analysis tools FluConvert and IniFlu, which exhibit high capacity and efficiency in data processing, analyzing, and combining large amounts of the most comprehensive influenza viral information retrieved from different public domain databases without making any modifications on downloaded genetic information. These tools not only provide a versatile and rapid platform for real-time analysis to determine consensus sequences, but also identify molecular markers with high pathogenicity in chickens as well as with interspecies transmission to humans. FluConvert and IniFlu are particularly useful in risk assessment by monitoring and analyzing the increasing trends of important amino acids of many animal influenza viruses with pandemic potential. While IniFlu is first designed for type A influenza viruses, the software can easily adapt to investigate other emerging viruses with appropriate modifications on the worksheet template. The software reported in this study provides a useful tool for rapidly identifying molecular signatures with virological, epidemiological and clinical significance.

## Conclusions

The rapid evolution of H5 AIVs in Asia has increased the threat in agricultural safety and human health. The timely monitoring in the changes of AIV that have increasing risk are important for public health-policy makers. FluConvert and IniFlu reported in this study are demonstrated for their efficiency in combining and analyzing virological, epidemiological and clinical information from different public domain databases. Finally, identification of polygenic signature for AIVs with high risk instead of variations at one single gene segment of influenza viruses will be beneficial to assist a better risk assessment to prevent pandemic influenza.

## Availability and requirements

**Project name**: FluConvert_IniFlu

**Project home page**: https://apps.flutures.com or https://github.com/chinrur/FluConvert_IniFlu

**Operating system(s)**: Microsoft Windows 10 or later version (64-bit)

**Programming language**: Batch (shell) scripts and VBA 7.1

**Other requirements**: Microsoft Office Excel 365 or Excel 2016 or later version (64-bit); Java 6 or higher version

**License**: MIT License.

**Any restrictions to use by non-academics**: No restrictions on use by non-academics.

## Data Availability

The H5N2 Dataset generated and analyzed during the current study are available in the NCBI-IVD (https://www.ncbi.nlm.nih.gov/genomes/FLU/) and GISAID-EpiFlu databases (https://www.gisaid.org/). These analysis tools are not intended for use in public domain but only for processing data retrieved from public databases. Their use would not breach the data access agreement of GISAID. Abiding by GISAID-EpiFlu Database Access Agreement, these tools will not generate new database for public access or make any annotation, correction, or modification of data submitted to GIASID EpiFlu Database.

## Abbreviations

AIV: avian influenza virus
CLI: command-line interface
EMBOSS: The European Molecular Biology Open Software Suite
FluCG: Flu Comparative Grouping
FluCS: Flu Cross-Segment alignment
GISAID: Global Initiative on Sharing All Influenza Data
GUI: graphical interface
HPAI: highly pathogenic avian influenza
IAV: influenza A virus
IRD: Influenza Research Database
IVD: Influenza Virus Database
MAFFT: multiple sequence alignment program with fast Fourier transform
NCBI: National Center for Biotechnology Information
ORF: open-reading-frame
VBA: Visual Basic Application
WHO: World Health Organization
